# Perinatal Exposure to a Diet High in Saturated Fat, Refined Sugar and Cholesterol Affects Behaviour, Growth, and Feed Intake in Weaned Piglets

**DOI:** 10.1371/journal.pone.0154698

**Published:** 2016-05-18

**Authors:** Caroline Clouard, Walter J. J. Gerrits, Bas Kemp, David Val-Laillet, J. Elizabeth Bolhuis

**Affiliations:** 1 Adaptation Physiology Group, Department of Animal Sciences, Wageningen University, Wageningen, The Netherlands; 2 Animal Nutrition Group, Department of Animal Sciences, Wageningen University, Wageningen, The Netherlands; 3 UR1341 ADNC, INRA, Saint Gilles, France; INIA, SPAIN

## Abstract

The increased consumption of diets high in saturated fats and refined sugars is a major public health concern in Western human societies. Recent studies suggest that perinatal exposure to dietary fat and/or sugar may affect behavioural development. We thus investigated the effects of perinatal exposure to a high-fat high-sugar diet (HFS) on behavioural development and production performance of piglets. Thirty-two non-obese sows and their piglets were allocated to 1 of 4 treatments in a 2 × 2 factorial design, with 8-week prenatal (gestation) and 8-week postnatal (lactation and post-weaning) exposure to a HFS diet (12% saturated fat, 18.5% sucrose, 1% cholesterol) or control low-fat low-sugar high-starch diets as factors. From weaning onwards (4 weeks of age), piglets were housed in group of 3 littermates (*n* = 8 groups/treatment) and fed *ad libitum*. After the end of the dietary intervention (8 weeks of age), all the piglets were fed a standard commercial diet. Piglet behaviours in the home pens were scored, and skin lesions, growth, feed intake and feed efficiency were measured up to 8 weeks after the end of the dietary treatment, *i*.*e*. until 16 weeks of age. At the end of the dietary treatment (8 weeks of age), response to novelty was assessed in a combined open field and novel object test (OFT/NOT). During the weeks following weaning, piglets fed the postnatal HFS diet tended to be less aggressive (p = 0.06), but exhibited more oral manipulation of pen mates (p = 0.05) than controls. Compared to controls, piglets fed the prenatal or postnatal HFS diet walked more in the home pen (p ≤ 0.05), and tended to have fewer skin lesions (p < 0.10). Several behavioural effects of the postnatal HFS diet depended on the prenatal diet, with piglets subjected to a switch of diet at birth being more active, and exploring feeding materials, pen mates, and the environment more than piglets that remained on the same diet. Behaviours during the OFT/NOT were not affected by the diet. The intake of the postnatal HFS diet drastically reduced feed intake, but improved feed efficiency up to 8 weeks after the end of the dietary intervention, *i*.*e*. 16 weeks of age (p < 0.0001 for both). Our study highlights the key role of prenatal and postnatal nutritional interactions for early behavioural development, and reveals programming effects of early life nutrition on voluntary feed intake of piglets later in life.

## Introduction

In the recent decades, modern human societies have been subjected to a rapid shift in diet composition, from food items high in complex carbohydrates and fibres to highly palatable and low-cost food, high in saturated fat, refined sugars and cholesterol. The increased availability and consumption of high-fat high-sugar (HFS) diets is a major public health concern, as it is accompanied by adverse health consequences, including an increased incidence of obesity, cardiovascular diseases and type-2 diabetes [1,2]. In addition, several human and animal studies suggest that the consumption of diets or beverages rich in fat and/or refined sugars affects behavioural and emotional processes, including anxiety/depression [3–5] and aggression [4,6,7]. In addition to the impact of dietary fat and refined sugars on behaviours, dietary cholesterol, another major component of the modern Western diet, has been found to affect aggression and social interactions in monkeys [8] and minipigs [9].

Inevitably, the rise in consumption of HFS diets in Western modern societies leads to an increased exposure to these dietary components during the perinatal developmental period, notably *via* the maternal diet, maternal milk or early life diet. It is now widely accepted that perinatal conditions, and notably prenatal and early life nutrition, can have long-lasting impacts on health and behaviours [10,11]. Accordingly, growing evidence indicates that perinatal exposure to high levels of dietary fat and/or sugars, often associated with maternal obesity, has strong programming effects on health [12,13] and behaviour of offspring in later life, as reviewed by Sullivan *et al*. [14]. In non-human male primates [15] and rats [16], increased aggressive behaviours have been reported in offspring of mothers fed a high-fat diet. In rats [17] [18,19], mice [20] and non-human primates [15], offspring exposed to a maternal high-fat diet also showed increased anxiety-like behaviours in a variety of tests later in life. On the other hand, some studies have reported reduced anxiety-related behaviours and motor activity, and increased exploration and social behaviours in rats exposed to a cafeteria diet (*i*.*e*. high in both refined sugars and saturated fat) *via* the maternal diet [21] or *via* the post-weaning diet [22].

Research in animal models has revealed the impact of perinatal nutrition on health and behaviours in later life. There is, however, a large variability in methodologies between studies, resulting in equivocal research findings. First, while the majority of studies have addressed the effects of diets enriched in fat alone, only a few studies have used diets enriched in both fat and refined sugars. Yet, in modern Western societies, excessive consumption of fat is frequently associated with the consumption of excessive amounts of refined sugars [23]. Furthermore, in most published studies, mothers became obese as a result of the long-term intake of a high-fat or high-energy diet, making it hard to disentangle the effects of the diet *per se* from those of the maternal health status [14,24]. In addition, very little is known on the effects of maintaining the offspring on the high-fat and/or high-sugar diet after weaning, which would be highly relevant for human nutrition, since the early diet of children often resembles their parents’ eating habits [25,26]. Finally, as far as we know, no study has investigated the differential impact of prenatal *vs*. postnatal nutrition on behavioural development.

While the large majority of the studies performed on rodents have yielded substantial scientific knowledge for future research on perinatal nutrition, the metabolic and physiological differences between rodents and humans complicate the translation of research findings into applications in human health and nutrition [27]. The pig is a highly social species that shares several similarities with humans in terms of intestinal and brain anatomy and physiology [28,29]. The pig therefore emerges as a pertinent animal model to study the influence of early nutrition on spontaneous behaviours, *e*.*g*. aggression and social interactions, and responses to specific stimuli during behavioural tests, *e*.*g*. anxiety- or fear-related behaviours.

We investigated the effects of prenatal and/or postnatal exposure to a HFS diet on the behavioural development of piglets. To that aim, the effects of prenatal and/or postnatal exposure to a diet enriched in saturated fat, sucrose, and cholesterol was compared with exposure to control diets, low in these components but high in starch. We hypothesized that 8-week prenatal (through the maternal diet during gestation) and/or 8-week postnatal (through maternal milk during the suckling period and piglet feed after weaning) exposure to the HFS diet would have adverse effects on behaviours, skin lesions as an indicator of aggression [30], and emotional reactivity (*i*.*e*. anxiety- or fear-related behaviours) of piglets after weaning. Behavioural recordings are discussed in relation to measurements of growth, feed intake and feed efficiency.

## Materials and Methods

The Animal Care and Use Committee of Wageningen University has approved this experiment.

### Animals and housing

A total of 32 multiparous sows and their litters (Tempo × Topigs 20) from the Swine Innovation Centre of Wageningen UR (VIC, Sterksel, The Netherlands) were used. The experiment was carried out in 2 successive replicates, with 16 sows per replicate. During gestation, the sows were housed in groups of 4 or 5 sows in pens (12 m^2^) with individual feeding stalls (230 cm × 72 cm). Room temperature was maintained at 18–20°C. One week before the expected parturition date, sows were housed in individual farrowing pens (240 cm × 180 cm) distributed in 2 rooms, and with partly solid (65%) and partly slatted (35%) floors. Distribution of sows over and within the 2 rooms was balanced for treatment. A jute bag was available in the pen until after parturition to be used as nesting material. During the suckling period, sows were confined in a farrowing crate, and a creep area with plastic flooring and a heating lamp above it was available for the piglets. Room temperature was maintained at a minimum of 23°C. Piglets remained in the sow’s farrowing pen with littermates until weaning. Within 3 days after birth, if needed, piglets were cross-fostered within dietary treatment groups to balance litter sizes. At 4 days of age, piglets were ear-notched for identification, received iron injections and had their tails docked. During gestation and lactation, the sows had *ad libitum* access to water and rooms had a natural light-dark cycle.

At weaning (*i*.*e*. 4 weeks of age), 3 female piglets with a birth weight the closest to the average birth weight of the females of the litter were selected per litter. Piglets with a birth weight less than 600 g and with a history of leg problems or long-term medication were excluded from selection. After weaning, the selected piglets were transported to the experimental farm Carus (Wageningen UR, The Netherlands) and housed in groups of 3 littermates in pens (280 cm × 180 cm) distributed in 2 identical and adjacent rooms. Pens were enriched with wood shavings and equipped with a chain with screws attached to it as a toy. Distribution of piglets over and within the 2 rooms was balanced for treatment. Seventy Litres of fresh wood shavings and, at the end of the dietary treatment (*i*.*e*. 8 weeks of age), 50 g of straw were added daily in the pens. Piglets had *ad libitum* access to a feeder and a drinking nipple. A heating lamp was provided for the first 4 post-weaning weeks. During the first 2 post-weaning weeks, room temperature was maintained at a minimum of 25°C. Room temperature was then decreased to 22°C for the 2 following weeks, and to 20°C for the rest of the experiment. Lights were on from 7:00 h to 19:00 h, and pop radio music was broadcasted live in the rooms during light time.

### Dietary treatments

Four experimental diets were formulated (Table 1), with 3 control diets: for the sows, a standard gestation diet and a standard lactation diet; for the piglets, a standard starter diet; and a HFS diet for both sows and piglets, *i*.*e*. an “all-in-one” diet high in saturated fat (lard), sucrose, and cholesterol. All the sows and piglets were fed standard commercial diets before the start of the experiment (*i*.*e*. before day 59 of gestation) and/or after the end of the dietary treatment (*i*.*e*. 8 weeks of age). All diets were provided as pellets. The control diets were formulated to meet or exceed the recommendations for animals’ requirements by CVB [31].

**Table 1:** Ingredient and nutrient composition of the experimental diets^1^.

	Gestation diet	Lactation diet	Starter diet	HFS diet
***Ingredient composition (%)***				
Barley	34.0	26.1	24.2	15.0
Wheat	19.9	29.0	23.2	16.9
Wheat bran	15.0	10.0	‒	5.00
Soybean meal	11.0	14.0	22.6	10.0
Maize	10.0	12.0	25.0	‒
Dehydrated sugarbeet pulp	5.00	‒	‒	‒
Sugarbeet molasses	‒	3.00	‒	‒
Vegetable fat	2.00	‒	‒	‒
**Sucrose**	**‒**	**‒**	**‒**	**18.5**
**Animal fat (Lard)**	**‒**	**‒**	**‒**	**12.0**
**Cholesterol**	**‒**	**‒**	**‒**	**1.00**
Peas	‒	‒	‒	7.00
Potato protein	‒	‒	‒	5.00
Soybean hulls	‒	‒	‒	5.00
Calcium carbonate	1.20	1.15	1.70	1.50
Dicalcium phosphate	0.85	1.50	‒	1.60
Monocalcium phosphate	‒	‒	1.35	‒
Premix	0.50	0.50	0.50	0.50
Sodium chloride	0.45	0.45	0.40	0.60
Acidifying agent	0.10	0.10	0.10	‒
Phytase	0.01	0.01	0.01	‒
Sodium bicarbonate	‒	‒	‒	0.40
Soy oil	‒	2.00	0.45	‒
L-lysine HCl	‒	0.20	0.34	‒
L-threonine	‒	0.04	0.08	‒
DL-Methionine	‒	‒	0.11	‒
L-tryptophan	‒	‒	0.02	‒
***Nutrient composition (g/kg DM)***^2^				
**Sugar**	**46.0**	**60.0**	**45.0**	**225**
**Crude fat**	**51.0**	**50.0**	**37.0**	**147**
**Starch**	**420**	**439**	**466**	**238**
**Crude protein**	**166**	**178**	**207**	**171**
Crude fibre	59.0	43.0	35.0	48.0
Crude ash	60.0	64.0	66.0	61.0
NDF	210	167	127	128
ADF	74.0	56.0	46.0	59.0
NSP	266	217	187	169
fNSP	149	120	121	100
Lysine^3^	6.00	8.20	11.3	8.60
Methionine + cystine^3^	4.80	5.00	6.90	4.80
Threonine^3^	4.60	5.40	7.00	5.80
Tryptophan^3^	1.70	1.80	2.30	1.80
DM (g/kg)	878	873	876	918
**NE (MJ/kg DM)**	**10.7**	**10.9**	**11.0**	**13.2**

^1^ DM: dry matter; NDF: neutral detergent fibre; ADF: acid detergent fibre; (f)NSP: (fermentable) non-starch polysaccharides; NE: net energy.

^2^ Based on calculated values [31].

^3^ Standardized ileal digestible.

During the last 8 weeks of gestation, half of the sows received the HFS diet and the other half the standard gestation diet. Starting on the day of parturition, half of the sows of each group received the HFS diet and the other half the standard lactation diet during the 4 weeks of lactation. Starting on the day of weaning, the piglets were fed the HFS diet or the standard starter diet for 4 weeks, so that they remained on the same treatment as their mothers during lactation. Consequently, piglets were allocated to one of 4 dietary treatments in a 2 × 2 factorial design, with 8-week prenatal/ 8-week postnatal exposure to the HFS or control diets (CON) as factors: HFS/HFS, CON/HFS HFS/CON, and CON/CON treatment. Allocation to the treatments was balanced for sow parity, and treatments were evenly distributed between replicates, as well as within and between the rooms during gestation, lactation and after weaning.

During gestation and lactation, the sows were restrictedly fed twice daily to meet the normal energy recommendations for pregnant and lactating sows. As the organoleptic properties of the HFS diet strongly differed from that of the gestation and lactation diets, gradual feed transitions over 4 days (*i*.*e*. 25%, 50%, 75%, 100% of the HFS diet in the total feed ration and *vice versa*) were done to prevent neophobic responses of the sows when exposed to a change in diet. Additionally, from 16 days before weaning, piglets also received the HFS or control starter diet (0.12 kg/piglet/day) as a creep feed to facilitate the transition to solid feed after weaning. Piglets were fed *ad libitum*.

### Measurements

#### Weighing, feed intake and skin lesions

Piglets were weighed at birth. Piglets and feed refusals were weighed on 3 occasions, *i*.*e*. on the day of weaning (4 weeks of age), at the end of the dietary treatment (8 weeks of age), and 8 weeks later (16 weeks of age). Average daily gain (ADG, kg/day), average daily feed intake (ADFI, kg of feed/day) and gain to feed ratio (G:F, *i*.*e*. a measure of feed efficiency) were calculated per pen from weaning to the end of the treatment and from the end of the treatment to 8 weeks later. Net energy (NE, MJ) intake and the gain:NE intake ratio were also presented. On the day of weaning, and 7, 14 and 21 days later, the number of skin lesions was counted on the front, middle and rear parts of the body of standing piglets in the home pen, according to the procedure of Turner *et al*. [30]. Scratches (*i*.*e*. superficial lesions) and deep wounds (*i*.*e*. haemorrhages) were differentiated.

#### Behavioural observations in the home pen after weaning

On the day of weaning, and 15 and 27 days after weaning, behaviours of weaned piglets were scored live using 2-min instantaneous scan sampling for 6 h/day during 6 sessions of 1 h (8:00–9:00 h, 9:15–10:15 h, 10:30–11:30 h, 14:00–15:00 h, 15:15–16:15 h, 16:30–17:30 h). The behaviours (S1 Table) were recorded using a Psion hand-held computer with the Observer 5.0 software package (Noldus Information Technology B.V., Wageningen, The Netherlands).

#### Feed preference tests

The day after the end of the dietary treatment (*i*.*e*. 8 weeks of age) and 8 weeks later, the piglets were subjected to a two-choice feed preference test. The control starter diet and the HFS diet were distributed in excess to estimated intake in two adjacent feeders at 9:00 h. Feed refusals were weighed at 16:00 h, *i*.*e*. after 7 h of access to the feed. If needed, feed was added in the feeders to ensure that no feeder was emptied throughout the night. Feed refusals were weighed again at 9:00 h the next day, *i*.*e*. after 24 h of access to the feed. Assignment of the diets to the left and right feeders was counterbalanced across pens, dietary treatment and testing days to avoid any laterality bias.

#### Novel environment and novel object test

Starting 5 days after the end of the dietary treatment, 2 piglets per pen were individually subjected to a combined open field (OFT) and novel object test (NOT) [32,33]. Before the tests, the piglets were habituated to the experimenters in their pens for 7 days. Each day, the experimenters entered each pen in random order, and stayed in each pen for approximately 5 min. First the experimenter sat in the pen (d 1 and 2), squatted (d 2 and 3), stood still in the pen (d 3 to 4), and finally walked in the pen (d 5 to 7). Each day, the experimenter allowed the piglets to approach spontaneously and tried to touch their nose, head, and body. At the end, the experimenter could walk at a normal pace around the pen and touch all piglets without signs of stress.

**Fig 1:**
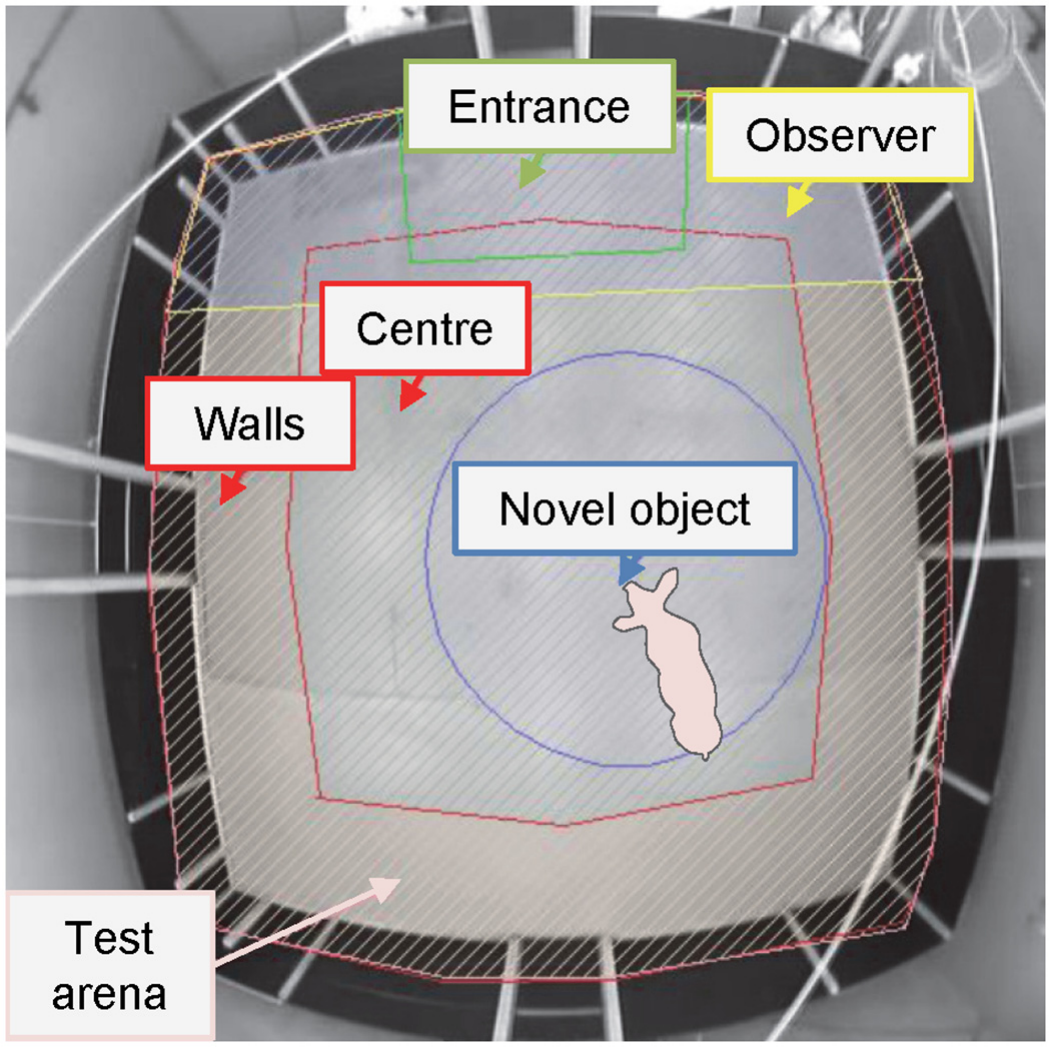
Zones in the open field and novel object tests. The zones were defined using the Ethovision XT 10 video tracking software to perform *a posteriori* analyses of locomotion patterns in the arena.

The tests were carried out on 2 consecutive days with half of the pigs being tested on day 1 and the other half on day 2, balanced for treatment. The test arena was 5.27 x 5.25 m with wooden black walls (1.2 m high) and concrete floor. The arena was located in a room adjacent to the piglets’ home pens. The OFT started when the piglet had entered the arena with all 4 hooves and the door was closed. After 5 min, the NOT started by dropping a metal bucket from the ceiling until it touched the floor at the centre of the test arena, resulting in a sudden noise. The bucket was then left on the floor for 5 min. After each test, faeces were removed from the test arena, which was cleaned with water and cleanser, and dried with towels. The order of testing was balanced for treatment and room. During the tests, behaviours and postures (S2 Table) were scored live using Psion hand-held computers with the Observer 5.0 software package (Noldus Information Technology, Wageningen, The Netherlands). The tests were also recorded on video to perform *a posteriori* analyses of locomotion patterns in the arena using the Ethovision XT 10 video tracking software (Noldus Information Technology B.V., Wageningen, The Netherlands). The variables calculated in Ethovision were: the time spent in the centre, walls, observer, entrance and novel object zones (Fig 1), the latencies (s) to enter the centre and novel object zones, and the total distance (m) covered during the tests.

#### Saliva sampling and cortisol analysis

The day before the end of the dietary treatment, the piglets were habituated to the saliva collection procedure: they were allowed to chew on 2 cotton buds simultaneously for 1 min at least twice during the morning. Saliva samples were then collected at the end of the dietary treatment and 8 weeks later to determine basal cortisol levels. The day of the OFT/NOT, saliva samples were collected from each piglet in the home pen 10 min before (t_0_), and 15, 30 and 60 min after the piglet entered the test arena. Saliva was systematically sampled in the morning, before feeding. On the days of sampling, saliva was collected by allowing the pigs to chew on 2 cotton buds simultaneously until they were thoroughly moistened. The cotton buds were placed in plastic tubes (2 buds per tube, Sarstedt, Etten-Leur, The Netherlands) directly after sampling and stored on ice until all samples were taken. Then, samples were centrifuged at 1560 × g for 10 min at 4°C and stored at -20°C until further analyses. Salivary cortisol concentrations (ng/mL) were measured in duplicate using a radioimmunoassay kit (COAT-A-COUNT^®^, Siemens Healthcare Diagnostics, Los Angeles, USA) modified and validated for pigs [34], with an intra-assay CV of 4% and an inter-assay CV of 5%.

### Statistical analyses

Statistical analyses were conducted using SAS version 9.1.3 (Statistical Analysis Software; SAS Institute, Cary, NC, USA). If the model residuals were not normally distributed, the statistical analyses were performed on (arcsin) square root or logarithmically transformed data. Two pens were excluded from the study because of a methodological error in replicate 2, resulting in *n* = 8 pens for CON/HFS and HFS/CON treatments, and *n* = 7 pens for HFS/HFS and CON/CON treatments. Data are presented as (untransformed) means ± SEMs. The upper limit for the statistically significant effect was set at p ≤ 0.05, with 0.05 < p ≤ 0.10 considered as a trend.

#### Effects of dietary treatment

Litter size at farrowing was analysed using a mixed model with prenatal diet and replicate as fixed effects. BW of piglets was averaged per pen. BW at birth was analysed using a mixed model with prenatal diet and replicate as fixed effects. BW, ADG and ADFI after weaning were analysed using mixed models with prenatal diet, postnatal diet, their interactions and replicate as fixed effects. BW at birth was included as covariate for BW at weaning. BW at weaning was included as covariate for BW after weaning, ADG, G:F and ADFI. Including BW at birth as a covariate for BW at weaning, and BW at weaning as a covariate for BW, ADG, G:F and ADGI after weaning did not change the results. Piglet behaviours in the home pen after weaning were averaged per pen and per day. Weaning is a major stressor for piglets, and the behaviours on the day after weaning may therefore reflect the response of piglets to this stressor rather than display their normal time budget. Indeed, behaviours on this day differed profoundly from behaviours 15 and 27 days later, and were therefore analysed separately. Behaviours on the day after weaning were analysed using a mixed model that included the fixed effects of prenatal diet, postnatal diet, their interactions and replicate. Behaviours at 15 and 27 days after weaning were analysed using a repeated mixed model with prenatal diet, postnatal diet, day, their interactions and replicate as fixed effects. As preliminary analyses showed that the number of skin lesions on the day of arrival to the home pen significantly differed from the number of lesions 7, 14 or 21 days after weaning, these data were analysed separately. The numbers of skin lesions the day of weaning were analysed using a mixed model that included the fixed effects of prenatal diet, postnatal diet, their interactions and replicate. Skin lesions at 7, 14 and 21 days after weaning were analysed using a repeated mixed model with prenatal diet, postnatal diet, day, their interactions and replicate as fixed effects. Piglet nested within pen, prenatal diet, postnatal diet and replicate was the experimental unit. In each treatment group (HFS/HFS, CON/HFS, HFS/CON, CON/CON), intake of the control starter diet during the two-choice feed preference tests was compared with the intake of the HFS diet by paired Student’s *t* tests. Salivary cortisol levels were averaged per pen and per sampling time. A mixed repeated model was used to estimate the fixed effect of prenatal diet, postnatal diet, sampling time, their interactions and replicate on salivary basal cortisol levels (at the end of the treatment and 8 weeks later), or cortisol levels before and after the OFT/NOT (before, and 15, 30 and 60 min after testing). Pen nested within prenatal diet, postnatal diet and replicate was the experimental unit.

#### Principal component analysis

A Principal Component Analysis (PCA) was conducted on the variables from the OFT/ NOT to examine whether variation in behavioural responses of the pigs (*n* = 63 piglets in total) could be summarized in a limited number of factors. In both the OFT and NOT, ‘jumping’, ‘comfort behaviours’, ‘sitting’, ‘lying’ and ‘barks’ hardly occurred, and were excluded from the analyses. ‘Nosing floor’, ‘rooting floor’, ‘nosing walls’ and ‘rooting walls’ were pooled as ‘exploring arena’. ‘Touching bucket’, ‘exploring bucket’ and ‘chewing bucket’ were pooled as ‘exploring bucket’. ‘Slow approach’ and ‘fast approach’ were pooled as ‘approaching bucket’. ‘Urinating’ and ‘defecating’ were pooled as ‘eliminating’. ‘Squeals’, ‘grunt squeals’ and ‘screams’ were pooled as ‘high-pitched vocalisations’, while ‘long grunts’, and ‘short grunts’ were pooled as ‘low-pitched vocalisations’. All variables were subjected to a general linear model with replicate as fixed effect to obtain residuals used for the PCA. After extraction, principal components were scaled by their standard deviations (square roots of associated Eigenvalues) and subjected to orthogonal rotation (varimax) to obtain independent factors. The factors retained from the PCA were analysed using a mixed model with prenatal diet, postnatal diet and their interactions as fixed effects, and pig as the experimental unit.

## Results

### Body weight and feed intake

Initial litter size was not affected by the prenatal diet (HFS: 15.38 ± 0.64 piglets, CON: 15.73 ± 0.62 piglets; F_1, 30_ = 0.08, p = 0.78). Data of piglets’ growth, feed intake and feed efficiency are presented in Table 2. The prenatal diet did not affect piglet BW at birth (HFS: 1.48 ± 0.05 kg, CON: 1.46 ± 0.06 kg). The day of weaning (*i*.*e*. 4 weeks of age), the piglets exposed to the prenatal HFS diet weighed more than the control piglets (HFS: 8.65 ± 0.16 kg, CON: 8.18 ± 0.19 kg).

**Table 2:** Body weight and average daily feed intake of piglets after weaning.

Prenatal diet	Control		HFS		p-values		
Postnatal diet	Control	HFS	Control	HFS	Prenatal	Postnatal	Prenatal × Postnatal
***Body weight (kg)***							
Birth	1.41 ± 0.06	1.50 ± 0.09	1.49 ± 0.09	1.48 ± 0.07	0.71	‒	‒
Weaning (4 weeks of age)	7.94 ± 0.34	8.43 ± 0.16	8.71 ± 0.29	8.60 ± 0.15	**0.04**	0.50	0.28
End treatment (8 weeks of age)	19.2 ± 1.13	15.9 ± 0.46	19.6 ± 0.91	16.3 ± 0.37	0.67	**< 0.0001**	0.67
8 weeks later (16 weeks of age)	73.0 ± 1.85	65.6 ± 1.66	73.8 ± 2.35	67.6 ± 1.93	0.94	**0.0004**	0.56
***Average daily gain (kg/day)***							
Weaning-End of treatment	0.36 ± 0.03	0.26 ± 0.02	0.39 ± 0.03	0.27 ± 0.01	0.75	**< 0.0001**	0.67
End treatment-8 weeks later	0.96 ± 0.03	0.89 ± 0.02	0.97 ± 0.03	0.92 ± 0.03	0.80	**0.02**	0.62
***Average daily feed intake (kg/day)***							
Weaning-End of treatment	0.58 ± 0.04	0.53 ± 0.03	0.62 ± 0.04	0.53 ± 0.03	0.82	**0.05**	0.75
End treatment-8 weeks later	2.11 ± 0.09	1.65 ± 0.06	2.07 ± 0.13	1.70 ± 0.06	0.49	**< 0.0001**	0.44
***Gain to feed ratio (kg weight/kg feed)***							
Weaning-End of treatment	0.67 ± 0.01	0.50 ± 0.03	0.63 ± 0.03	0.52 ± 0.03	0.52	**< 0.0001**	0.26
End treatment-8 weeks later	0.43 ± 0.02	0.51 ± 0.01	0.45 ± 0.01	0.51 ± 0.01	0.20	**< 0.0001**	0.58

Data are averaged per pen and presented as means ± SEMs. Significant main effects of prenatal diet, postnatal diet or their interactions are indicated in **bold** (p ≤ 0.05) and trends are indicated in ***bold italics*** (0.05 < p ≤ 0.10).

At the end of the treatment (8 weeks of age), the piglets exposed to the postnatal HFS diet weighed significantly less than the control piglets (HFS: 16.06 ± 0.29 kg, CON: 19.38 ± 0.69 kg). From weaning to the end of the treatment, the piglets exposed to the postnatal HFS diet had a lower ADG (-30%, HFS: 0.27 ± 0.01, CON: 0.39 ± 0.02 kg/day), ADFI (-12%, HFS: 0.53 ± 0.02 kg/day, CON: 0.60 ± 0.03 kg/day), and G:F (HFS: 0.51 ± 0.02 kg/kg feed, *i*.*e*. ~0.42 kg/10 MJ NE, CON: 0.65 ± 0.02 kg/kg feed, *i*.*e*. ~0.68 kg/10 MJ NE) compared to the control piglets. The piglets exposed to the postnatal HFS diet seemed to have higher daily NE intake than the control piglets (HFS: ~6.42 MJ NE/day, Control: ~5.78 MJ NE/day).

Eight weeks after the end of the treatment (16 weeks of age), the piglets exposed to postnatal HFS diet weighed significantly less than the control piglets (HFS: 66.54 ± 0.29 kg, CON: 73.42 ± 1.47 kg). From the end of the treatment to 8 weeks later, the piglets exposed to the postnatal HFS diet had a 20% lower ADFI (HFS: 1.68 ± 0.04, CON: 2.09 ± 0.08 kg/day), but only 7% lower ADG (HFS: 0.90 ± 0.02, CON: 0.97 ± 0.02 kg/day), resulting in an increased G:F (HFS: 0.51 ± 0.00, CON: 0.44 ± 0.01) compared to the control piglets. The prenatal diet or its interactions with the postnatal diet did not affect piglet BW, ADG, ADFI or G:F after weaning.

### Skin lesions

#### Day of weaning

**Fig 2:**
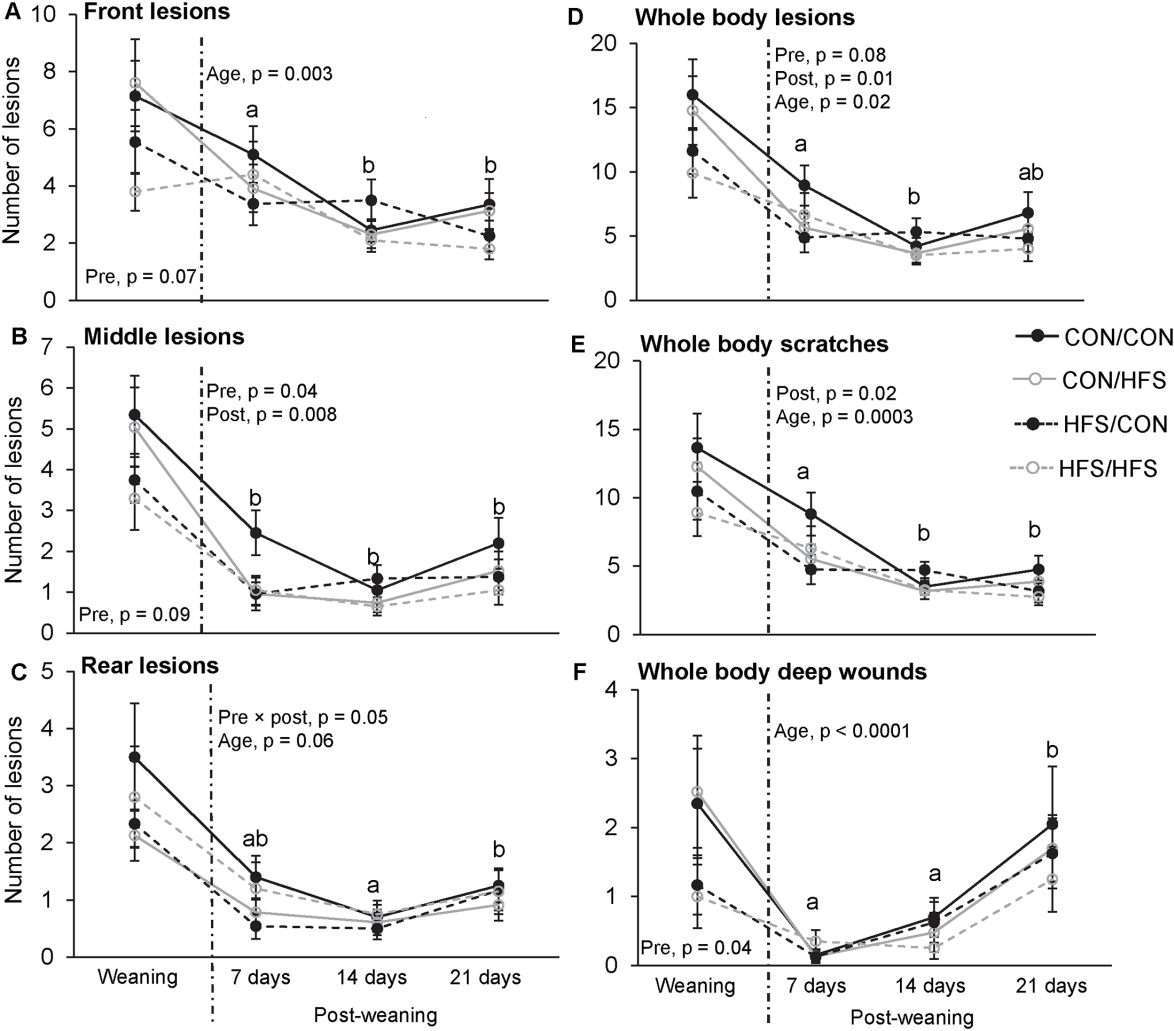
Skin lesions of piglets after weaning. (A) Total lesions, (B) total scratches and (C) total deep wounds on the whole body, and total lesions in the (D) front, (E) middle or (F) rear part of the body. Piglets were exposed to a HFS diet or a control (CON) diet 8 weeks before and/or 8 weeks after birth. Data on the day of weaning (left of the dotted line) were analysed separately from data on 7, 14 and 21 days after weaning (right of the dotted line). Different letters indicate a significant time effect between 7, 14 and 21 days after weaning (p ≤ 0.05).

The day of arrival to the post-weaning pen, the piglets exposed to the prenatal HFS diet tended to have fewer skin lesions on the front (Fig 2A) and middle parts (Fig 2B) of the body than the control piglets. The piglets exposed to the prenatal HFS diet also had fewer deep wounds on the whole body than the control piglets (Fig 2F). The postnatal diet did not affect body lesions on the day of weaning.

#### Post-weaning weeks

The weeks following weaning, both the postnatal, and to a lesser extent, prenatal HFS diet decreased the total number of skin lesions on the whole body (Fig 2D), which was mostly due to fewer scratches on the whole body (postnatal diet effect, Fig 2E), and/or fewer lesions on the middle part of the body (postnatal and prenatal diet effect, Fig 2B). The number of skin lesions on the rear part of the body was also affected by a prenatal diet × postnatal diet interaction, with the CON/HFS-piglets having fewer skin lesions on the rear part of the body than the CON/CON-piglets (Fig 2C).

### Behavioural observations

#### Day after weaning

The prenatal diet, postnatal diet and their interactions did not affect piglet behaviours on the day after weaning, with the exception that the piglets exposed to the postnatal HFS diet spent more time mounting pen mates than the control piglets (Table 3), and a tendency for prenatal diet × postnatal diet interaction effects for time spent on maintenance behaviours, standing and exploring pen mates. Post hoc tests did not reveal significant pairwise differences.

**Table 3:** Behavioural activity of piglets in the home pen the day after weaning.

Prenatal diet	Control		HFS		p-values		
Postnatal diet	Control	HFS	Control	HFS	Prenatal	Postnatal	Prenatal × Postnatal
***Inactive behaviours (%)***							
Lying	55.0 ± 3.98	55.0 ± 2.77	59.0 ± 3.55	48.0 ± 2.59	0.66	0.11	0.12
Sitting/kneeling	1.24 ± 0.27	1.00 ± 0.46	0.93 ± 0.19	1.11 ± 0.30	0.76	0.93	0.51
Standing	6.85 ± 0.92	6.16 ± 0.55	5.35 ± 0.47	7.51 ± 1.07	0.92	0.34	***0*.*06***
***Locomotion behaviours (%)***							
Walking	1.77 ± 0.51	2.41 ± 0.71	1.88 ± 0.46	2.22 ± 0.40	0.92	0.26	0.53
***Feed-related behaviours (%)***							
Ingesting feeding materials	6.48 ± 1.10	6.16 ± 1.09	5.49 ± 0.59	7.01 ± 0.91	0.94	0.54	0.33
Exploring feeding materials	1.34 ± 0.27	1.06 ± 0.34	1.11 ± 0.29	1.27 ± 0.30	0.85	0.84	0.35
***Exploratory behaviours (%)***							
Exploring environment	21.0 ± 2.41	22.8 ± 1.53	20.9 ± 2.51	26.1 ± 1.24	0.44	0.10	0.40
***Social behaviours (%)***							
Aggressing pen mates	1.03 ± 0.38	0.76 ± 0.41	0.65 ± 0.29	1.53 ± 1.13	0.82	0.95	0.35
Manipulating pen mates	0.45 ± 0.10	0.44 ± 0.19	0.69 ± 0.20	0.56 ± 0.08	0.13	0.50	0.63
Exploring pen mates	2.17 ± 0.27	1.39 ± 0.33	1.37 ± 0.34	1.75 ± 0.26	0.48	0.52	***0*.*08***
Mounting pen mates	0.05 ± 0.03	0.37 ± 0.19	0.09 ± 0.05	0.19 ± 0.10	0.64	**0.05**	0.29
***Playing behaviours (%)***							
Social, non-social and substrate playing	1.77 ± 0.55	2.04 ± 0.33	1.99 ± 0.60	1.72 ± 0.51	0.79	0.88	0.52
***Other (%)***							
Maintenance	0.40 ± 0.15	0.23 ± 0.12	0.25 ± 0.10	0.58 ± 0.24	0.35	0.47	***0*.*07***
Eliminating	0.16 ± 0.11	0.07 ± 0.03	0.12 ± 0.07	0.21 ± 0.06	0.50	0.96	0.23

Data are presented as % of observation (means ± SEMs). Significant main effects of prenatal diet, postnatal diet or their interactions are indicated in **bold** (p ≤ 0.05) and trends are indicated in ***bold italics*** (0.05 < p ≤ 0.10).

#### Post-weaning weeks

Behavioural activity in the home pen 15 and 27 days after weaning is presented in Table 4. As no interactions between prenatal diet or postnatal diet and time were found, data were averaged over both observation days. The weeks following weaning, the time spent lying and sitting/kneeling inactive was affected by prenatal diet × postnatal diet interactions. Post hoc comparisons showed that the CON/CON-piglets tended to spend more time lying inactive than the HFS/CON-piglets (p = 0.06), with levels of the CON/HFS- and HFS/HFS-piglets in between. The CON/CON-piglets and HFS/HFS-piglets spent more time sitting/kneeling than the CON/HFS-piglets (p = 0.01 for both), with levels of the HFS/CON-piglets in between. Standing inactive was increased by the postnatal HFS diet. Both the prenatal and postnatal HFS diet increased the time spent walking.

**Table 4:** Behavioural activity of piglets in the home pen 15 and 27 days after weaning.

Prenatal diet	Control		HFS		p-values		
Postnatal diet	Control	HFS	Control	HFS	Prenatal	Postnatal	Prenatal × Postnatal
***Inactive behaviours (%)***							
Lying	58.3 ± 3.41	53.0 ± 2.39	52.2 ± 1.77	56.0 ± 1.72	0.51	0.74	**0.05**
Sitting/kneeling	1.15 ± 0.28	0.43 ± 0.09	0.94 ± 0.25	1.13 ± 0.17	0.21	0.17	**0.02**
Standing	1.28 ± 0.28	2.54 ± 0.65	1.93 ± 0.47	2.60 ± 0.32	0.34	**0.01**	0.60
***Locomotion behaviours (%)***							
Walking	0.91 ± 0.22	1.20 ± 0.33	1.25 ± 0.45	1.71 ± 0.38	**0.03**	**0.05**	0.98
***Feed-related behaviours (%)***							
Ingesting feeding materials	11.9 ± 0.67	13.7 ± 1.40	12.7 ± 0.98	11.2 ± 0.82	0.28	0.87	**0.02**
Exploring feeding materials	0.71 ± 0.11	0.54 ± 0.08	1.00 ± 0.09	0.39 ± 0.10	0.94	**0.001**	**0.04**
***Exploratory behaviours (%)***							
Exploring environment	18.9 ± 2.07	20.7 ± 1.29	23.1 ± 1.68	19.1 ± 1.42	0.38	0.46	***0*.*07***
***Social behaviours (%)***							
Aggressing pen mates	0.26 ± 0.12	0.08 ± 0.03	0.22 ± 0.08	0.09 ± 0.07	0.50	***0*.*06***	0.72
Manipulating pen mates	1.16 ± 0.23	1.68 ± 0.31	1.44 ± 0.21	2.55 ± 0.91	0.22	**0.05**	0.76
Exploring pen mates	1.78 ± 0.40	1.91 ± 0.15	2.03 ± 0.22	1.34 ± 0.21	0.50	0.24	***0*.*08***
Mounting pen mates	0.08 ± 0.05	0.21 ± 0.06	0.13 ± 0.08	0.04 ± 0.02	0.25	0.44	***0*.*09***
***Playing behaviours (%)***							
Social, non-social and substrate playing	2.82 ± 0.61	3.20 ± 0.64	2.08 ± 0.20	2.98 ± 0.70	0.68	0.40	0.68
***Others (%)***							
Maintenance	0.18 ± 0.06	0.19 ± 0.08	0.19 ± 0.05	0.09 ± 0.03	0.30	0.30	0.12
Eliminating	0.59 ± 0.11	0.56 ± 0.11	0.63 ± 0.06	0.68 ± 0.07	0.37	0.87	0.53

Data are presented as % of observations (means ± SEMs). Within each treatment, data from day 15 and day 27 after weaning are averaged. Significant main effects of prenatal diet, postnatal diet or their interactions are indicated in **bold** (p ≤ 0.05) and trends are indicated in ***bold italics*** (0.05 < p ≤ 0.10).

The time spent exploring and ingesting feeding materials and exploring the environment was influenced by prenatal diet × postnatal diet interactions. Compared to the CON/HFS -piglets, the HFS/HFS-piglets spent less time ingesting feeding materials (p = 0.02). The HFS/HFS-piglets also spent less time exploring feeding materials than the HFS/CON- (p = 0.0004) and CON/CON-piglets (p = 0.02), while the HFS/CON-piglets explored feeding materials (p = 0.01) more than the CON/HFS-piglets. Numerical data suggest a lower time spent exploring the environment in the CON/CON- and HFS/HFS-piglets compared to the other groups.

The postnatal HFS diet tended to reduce time spent on aggression, but increased oral manipulation of pen mates. Trends were found for an effect of prenatal diet × postnatal diet interaction on the time spent mounting and exploring pen mates. Numerical data suggest less time spent mounting and exploring the pen mates in the CON/CON- and HFS/HFS-piglets compared to the other groups. The prenatal and postnatal diets did not affect the time spent on play behaviour, elimination or maintenance behaviour.

### Feed preference tests

**Fig 3:**
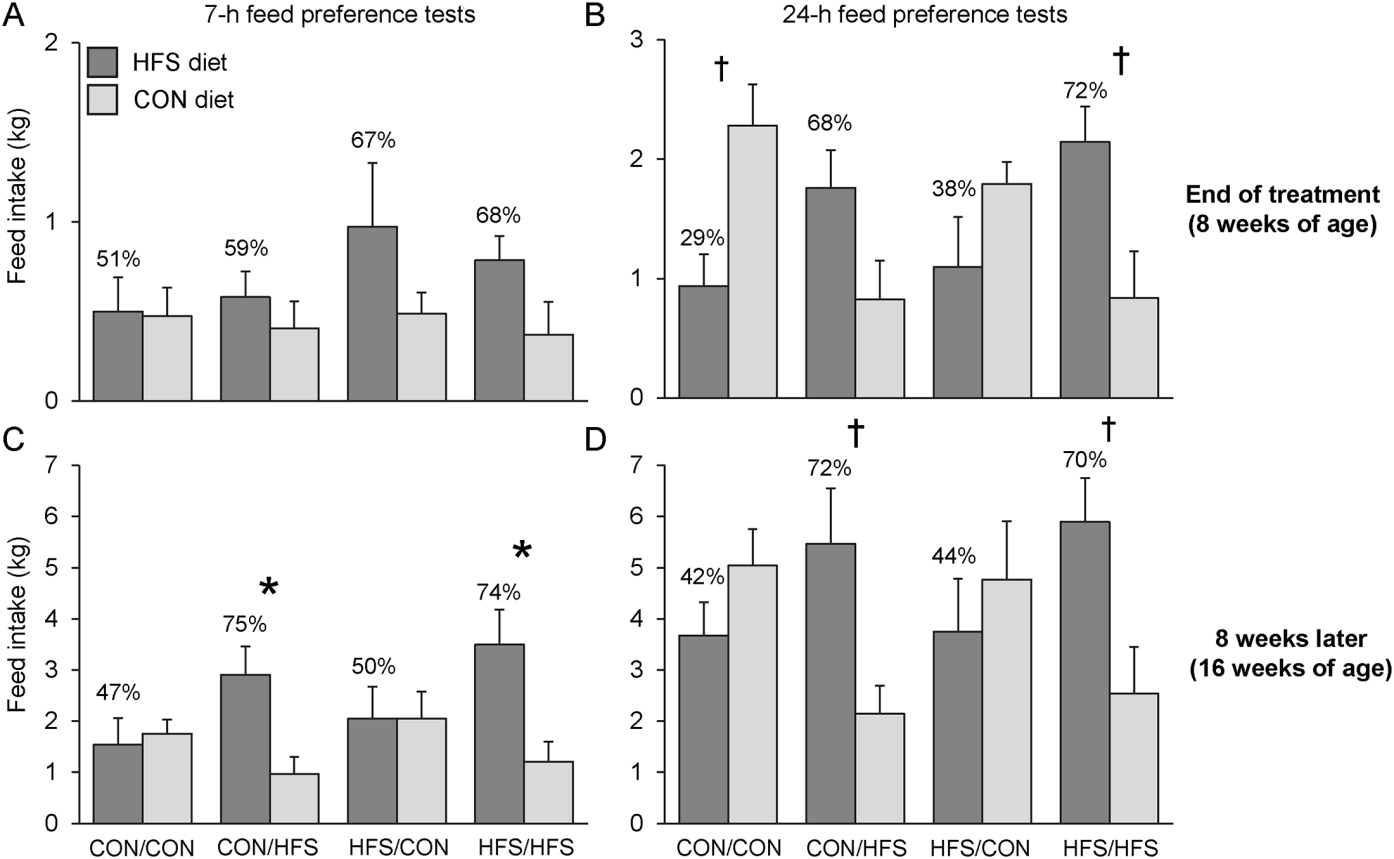
Feed intake (kg) during the feed preference tests. (A) 7‒h and (B) 24‒h feed preference tests done at the end of the dietary treatment, and (C) 7‒h and (D) 24‒h feed preference tests done 8 weeks later. During the tests, the piglets had the choice between the HFS diet and the control (CON) starter diet distributed in two feeders in the home pen. Percentages above bars indicate the intake of the HFS diet relative to total intake during the test. Symbols indicate significant differences between the absolute intake of the HFS *vs*. CON diet during the test: * *p* ≤ 0.05; † 0.05 < *p* ≤ 0.10.

Although the CON/CON‒piglets seemed to systematically prefer the control starter diet to the HFS diet, a statistical trend was only found for the 24‒h feed preference tests at the end of the treatment (p = 0.06; Fig 3). The CON/HFS‒piglets seemed to prefer the HFS diet to the control starter diet during both the 7‒h and 24‒h feed preference tests at the end of the treatment, but the preference did not reach the threshold for significance. Eight weeks later, however, the CON/HFS‒piglets tended to prefer the HFS diet to the control starter diet during both the 7‒h and 24‒h feed preference tests (p = 0.05 and p = 0.08, respectively). The HFS/CON‒piglets exhibited no consistent patterns of feed preferences during the tests (p > 0.10). The HFS/HFS‒piglets tended to prefer the HFS diet to the control starter diet during both the 7‒h and 24‒h tests at the end of the dietary treatment (p = 0.23 and p = 0.10, respectively) and 8 weeks later (p = 0.05 and p = 0.09, respectively).

### Principal component analysis

The variables (means ± SEMs) of the OFT/ NOT are presented in S3 Table. Seven factors were retained from the PCA (Eigenvalue > 1.2) and were labelled according to the variables loaded on the factor (Table 5). The factors explained 78% of total variance. The factor ‘Alert OFT & Vocalisations’ had high positive loadings for standing alert in OFT, and low- and high-pitched vocalisations in the OFT and NOT, and high negative loadings for time spent exploring the arena and walking in the OFT. The variables time spent in centre and novel object zones during the NOT loaded positively, and time spent in wall and observer zones during the NOT loading negatively on the ‘Location NOT’ factor. The factor ‘Locomotion NOT & Activity’ had high positive loadings for distance covered and time spent walking and exploring the arena during the NOT, and high negative loadings for time spent standing alert in the NOT and OFT. The time spent walking and the distance covered during the OFT loaded positively, and the time spent standing in the OFT loaded negatively in the ‘Locomotion OFT’ factor. The ‘Location OFT’ had high positive loadings for time spent in wall, observer and entrance zones, and a high negative loading for time spent in the centre of the OFT. The ‘Interactions with novel object’ factor had high positive loadings for the time spent walking, approaching the bucket and withdrawing from the bucket, and a high negative loading for standing in the NOT. Finally, the frequency of eliminating behaviours (*i*.*e*. defecating and urinating) in the OFT and NOT loaded positively, and the time exploring the novel object loaded negatively in the ‘Eliminating & Avoiding novel object’ factor. No trends or significant effects of prenatal or postnatal diet or their interaction were found on the retained PCA factors (Table 6).

**Table 5:** Loadings on the factors extracted by principal component analysis, after orthogonal rotation, of variables recorded on individual piglets.

Measures	Alert OFT & Vocalisations	Location NOT	Locomotion NOT & Activity	Locomotion OFT	Location OFT	Interactions with novel object	Eliminating & Avoiding novel object
***Open field test***							
Walking (%)	***-0*.*48***	0.26	0.30	**0.69**	-0.03	0.11	0.10
Standing alert (%)	**0.67**	-0.21	***-0*.*48***	0.01	-0.11	0.04	-0.22
Standing (%)	-0.14	-0.07	0.19	**-0.82**	0.25	-0.20	0.13
Exploring arena (%)	**-0.76**	0.08	0.38	-0.09	-0.05	0.08	0.24
Low-pitched vocalisations (freq)	**0.72**	0.16	0.01	0.38	0.14	0.00	0.03
High-pitched vocalisations (freq)	**0.64**	0.29	0.02	0.34	0.02	-0.12	0.21
Eliminating (freq)	0.19	0.08	0.06	‒0.01	0.14	‒0.04	***0*.*47***
Distance covered (m)	0.21	0.25	‒0.01	**0.74**	0.15	‒0.12	‒0.01
Latency to centre zone (s)	‒0.02	0.10	0.08	‒0.28	0.04	‒0.03	0.16
Time in the centre zone (%)	0.06	0.05	‒0.16	0.23	**‒0.65**	0.28	0.15
Time in wall zone (%)	0.06	0.12	0.01	‒0.03	**0.76**	‒0.23	‒0.01
Time in observer zone (%)	0.26	‒0.16	‒0.10	0.07	**0.73**	0.12	0.15
Time in entrance zone (%)	-0.03	‒0.21	‒0.23	0.02	***0*.*45***	0.18	0.28
***Novel object test***							
Walking (%)	0.19	‒0.04	**0.72**	-0.22	‒0.08	**0.50**	‒0.15
Standing alert (%)	‒0.08	‒0.10	**‒0.83**	0.16	0.01	0.02	‒0.02
Standing (%)	0.04	0.04	0.07	‒0.06	0.14	**‒0.67**	0.26
Exploring arena (%)	‒0.01	‒0.18	***0*.*48***	‒0.12	‒0.35	0.03	0.32
Low-pitched vocalisations (freq)	**0.74**	0.11	0.20	‒0.12	0.07	‒0.03	0.05
High-pitched vocalisations (freq)	**0.58**	0.24	0.16	‒0.08	0.06	‒0.14	0.11
Eliminating (freq)	‒0.07	‒0.04	0.19	‒0.21	0.01	‒0.09	**0.51**
Distance covered (m)	‒0.02	0.12	**0.50**	0.37	-0.21	‒0.06	0.20
Latency to centre zone (s)	‒0.03	0.25	0.16	‒0.26	‒0.05	0.12	‒0.15
Time in the centre zone (%)	0.12	**0.89**	‒0.05	0.11	‒0.13	0.13	0.13
Latency to novel object zone (s)	‒0.18	‒0.24	‒0.04	‒0.13	‒0.10	0.18	‒0.14
Time in novel object zone (%)	0.08	**0.87**	0.02	0.08	‒0.05	‒0.03	‒0.01
Time in wall zone (%)	‒0.13	**‒0.87**	0.03	‒0.03	‒0.13	0.01	0.04
Time in observer zone (%)	0.01	**‒0.59**	***0*.*48***	‒0.04	0.31	0.03	0.22
Time in entrance zone (%)	‒0.17	‒0.31	**0.55**	0.05	0.15	‒0.03	‒0.01
Approaching bucket (%)	‒0.08	0.17	0.13	‒0.02	0.00	**0.75**	‒0.03
Withdrawing from bucket (%)	‒0.12	‒0.06	‒0.01	0.07	‒0.04	**0.80**	0.19
Exploring bucket (%)	0.24	0.11	0.30	‒0.22	0.34	‒0.11	**‒0.59**
***Variance explained (%)***	**19.02**	**14.76**	**12.99**	**9.74**	**8.69**	**7.21**	**5.60**

Variables were recorded in 8-week-old piglets (*n* = 63) during the combined open field and novel object tests. Proportions of total variation explained by each factor are given. Loadings ≥ 0.40 or ≤ ‒0.40 are indicated in ***bold italics***, and loadings ≥ 0.50 or ≤ ‒0.50 are indicated in **bold**.

**Table 6:** Behavioural responses of piglets in the open field and novel object tests expressed as factor scores extracted by principal component analysis.

Prenatal diet	Control		HFS		p-values		
Postnatal diet	Control	HFS	Control	HFS	Prenatal	Postnatal	Prenatal × postnatal
Alert OFT & Vocalisations	0.29 ± 0.47	-0.01 ± 0.24	-0.31 ± 0.29	0.06 ± 0.31	0.38	0.96	0.32
Location NOT	0.09 ± 0.23	-0.26 ± 0.24	0.07 ± 0.17	0.01 ± 0.26	0.55	0.56	0.56
Locomotion NOT & Activity	0.37 ± 0.22	-0.12 ± 0.41	0.15 ± 0.27	-0.27 ± 0.32	0.67	0.12	0.82
Locomotion OFT	-0.15 ± 0.20	-0.18 ± 0.44	0.14 ± 0.19	0.08 ± 0.17	0.49	0.84	0.51
Location OFT	0.19 ± 0.23	-0.51 ± 0.30	0.14 ± 0.20	-0.06 ± 0.22	0.75	0.12	0.43
Interaction with novel object	-0.07 ± 0.45	-0.03 ± 0.21	0.30 ± 0.24	-0.12 ± 0.28	0.56	0.67	0.31
Eliminating & exploring novel object	0.13 ± 0.21	-0.03 ± 0.23	0.17 ± 0.23	-0.26 ± 0.25	0.93	0.38	0.58

Data are presented as means ± SEMs.

### Salivary cortisol analyses

**Fig 4:**
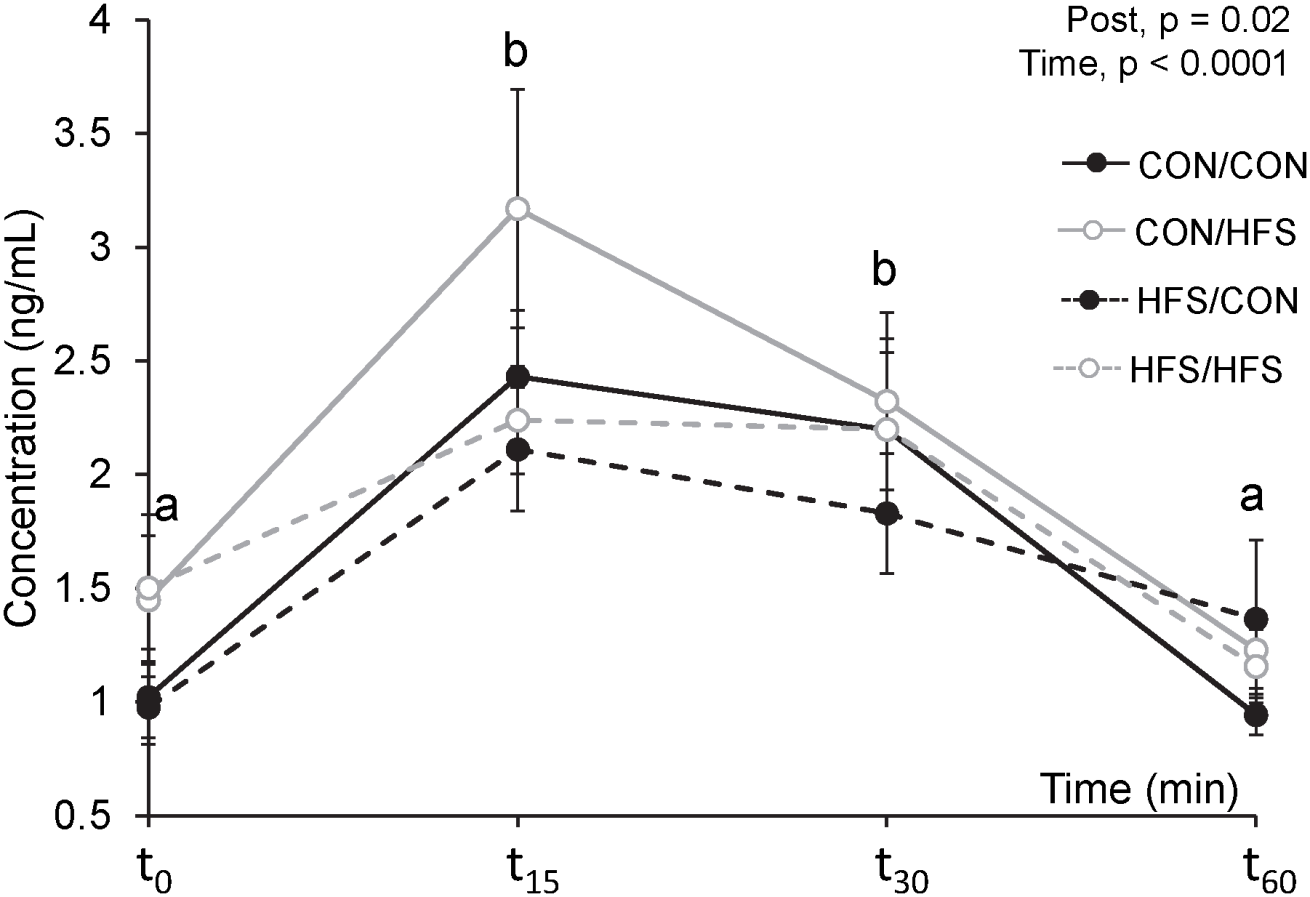
Salivary cortisol concentrations the day of the open field and novel object tests. Piglets were exposed to a HFS diet or a control (CON) diet 8 weeks before and/or 8 weeks after birth. Saliva samples were collected 10 min before (t_0_), and 15 (t_15_), 30 (t_30_) and 60 (t_60_) min after start of the test. Different letters indicate a significant time effect (p ≤ 0.05).

Compared to the prenatal control diet, the prenatal HFS diet tended to decrease basal salivary cortisol levels measured at the end of the treatment and 8 weeks later (HFS: 2.68 ± 0.20 ng/mL, CON: 2.26 ± 0.19 ng/mL, F_1, 27_ = 3.75, p = 0.06). No effects of the postnatal diet, time or their interactions were found on basal salivary cortisol levels (p > 0.10). A significant effect of time was found on salivary cortisol levels the day of the OFT/NOT (Fig 4), with a transient increase in concentration 15 min after the test and a return to basal levels 60 min after the test. The day of the OFT/NOT, the piglets exposed to the postnatal HFS diet had higher salivary cortisol levels than the control piglets (HFS: 1.92 ± 0.14 ng/mL, CON: 1.61 ± 0.12 ng/mL).

## Discussion

This study showed that late prenatal and/or early postnatal exposure to a diet enriched in saturated fat, sucrose, and cholesterol had no evident effects on anxiety- or fear-related behaviours in a novelty test, but impacted locomotion, social and exploratory behaviours, as well as long-term feed intake, growth, and feed efficiency in weaned piglets.

Both prenatal and postnatal exposure to the HFS diet affected piglet aggressive behaviour in the home pen after weaning. Piglets exposed to the HFS diet *before birth* had fewer skin lesions on the front and middle body parts, and fewer deep wounds on the whole body on the day of arrival to the post-weaning pen. Lesions on the front part of the body and on the flanks are valid indicators of reciprocal fighting [30], indicating that piglets fed the HFS diet *before birth* engaged less, and with lower intensity, in reciprocal fights in the farrowing crate and/or during transport in the truck. During the weeks following weaning, piglets exposed to the HFS diet *after birth* exhibited fewer aggressive behaviours, and had fewer skin lesions on the whole body than control piglets. Notably, compared to piglets not exposed to the HFS diet at all, piglets exposed to the HFS diet *only after birth* had fewer skin lesions on the rear part of the body, which reflect receiving un-retaliated aggression, *i*.*e*. ‘bullying’ behaviours [30]. This suggests that the postnatal HFS diet decreased not only ‘normal’ reciprocal aggression, but also aberrant bullying behaviours during the early post-weaning period. High-fat and high-sugar diets have been found to exert some “comforting effects” in stressful situations, with both animals and humans showing a tendency to consume this so-called “comfort food” following chronic stress [35]. We speculate that chronic exposure to the HFS diet, a palatable “comfort food”, may have alleviated the social tensions, and reduced the agonistic interactions after weaning, a stressful event in pig husbandry.

The intake of the HFS diet *after birth* affected other social interactions, with more mounting directly after weaning, and more manipulative behaviours directed at pen mates during the weeks following weaning. Mounting is a complex social behaviour, which has been linked not only with dominance and sexual behaviours [36], but also with motivation to play or explore, and a high state of arousal [37,38]. Manipulative behaviours (*e*.*g*. belly nosing, chewing tail or ears of pen mates) are known to represent re-directed exploratory or suckling behaviours in weaned piglets [39,40]. Although excessive occurrences of these behaviours may reflect poor adaptation to weaning [41], can cause severe skin injuries, and are not desirable on pig farms [42,43], their frequency remained very low in our study. The increase in mounting and, possibly, manipulative behaviours might thus reflect an enhanced motivation for social contact [44] rather than an increase in negative social interactions *per se*.

Prior studies have reported increased agonistic behaviours with a high-fat diet in non-human primates [15] and rats [4,16]. Others found fewer skin lesions, reduced aggression, and increased non-agonistic social interactions in juvenile minipigs fed a diet high in fat and cholesterol [9] or in juvenile monkeys fed a high-cholesterol diet [8]. We also reported decreased aggression with a high-cholesterol diet, suggesting that dietary cholesterol may have inhibitory effect on aggressiveness. Alternatively, we cannot rule out the possibility that the decrease in aggression was caused by high levels of sucrose, or the combination of high levels of fat and sugars in the HFS diet. Accordingly, Lalanza *et al*. [22] found increased social interactions in juvenile rats fed a cafeteria diet high in both fat and refined sugars for 8 weeks after weaning while intake of beverages high in sugars has been found to decrease aggression in adolescent humans [7].

We found no evidence of dietary effects on anxiety-related behaviours in the OFT/NOT, nor on the behavioural activity of piglets on the day directly after weaning. As stated earlier, weaning is a stressful event, and changes in behavioural activity (*e*.*g*. increased aggression, abnormal behaviours) are thought to reflect the piglet’s ability to cope with this stressful challenge. Taken together, our behavioural data do not support the postulate that a HFS diet would increase stress or anxiety-related behaviours, thus contradicting prior research in rodents and non-human primates [15,17–20]. Prior studies have confirmed that standing alert, high-pitched vocalisations, and defecations in novelty tests are related to negative emotional states in pigs [32,45]. This, together with the transient increased in salivary cortisol concentrations after the test, suggests that the combined OFT/NOT is a significant stressful event, and a relevant test to assess emotional reactivity in piglets. Discrepancies in results between studies may be related to differences in diet composition. While increased anxiety levels have been found in offspring from obese mothers fed high-fat diets [17–19], some studies have reported reduced anxiety-related behaviours in rats exposed to a maternal high-fat high-sugar cafeteria diet [21] or fed a cafeteria diet after weaning [22], suggesting that high-fat diets and diets combining high levels of fat and refined sugars may have contrasted effects on anxiety-related behaviours.

It is worth noting that, although the diet did not affect behaviours during the OFT/NOT, piglets exposed to the HFS diet *after birth* had higher salivary cortisol levels than control piglets on the day of testing. This difference in cortisol levels, however, was already observed at t_0_, *i*.*e*. basal level before testing, indicating that the high cortisol levels were likely not caused by an enhanced physiological response to acute stress, but rather by a chronic difference in basal cortisol levels. How the postnatal HFS diet affected cortisol levels and whether this was related to the presence of cholesterol, a cortisol precursor, in this diet, is unclear. On the other hand, piglets exposed to the HFS diet *before birth* tended to have reduced basal salivary cortisol levels after weaning compared to control piglets. Lower basal serum corticosterone levels have been found in rat offspring exposed to a maternal high-fat diet [17], while increased basal plasma corticosterone levels have been reported in female rats fed a post-weaning high-fat diet [46], and in male rats fed a post-weaning ‘junk food fatty diet’ [47]. These findings indicate that the programming effects of a prenatal high-fat diet on the hypothalamic-pituitary-adrenal axis responsivity may be different from that of a postnatal diet.

Both prenatal and postnatal exposure to the HFS diet increased the time spent walking in the home pen during the weeks following weaning, which contradicts prior findings in rats [17,20–22]. It is worth noting that, while we found increasedlocomotion in the familiar home pen, the aforementioned studies measured locomotion during novelty tests, in which locomotion may have been affected by stress [48]. Alternatively, it is possible that high-fat and high-fat/sugar diets exert differential effects on locomotion, as supported by prior studies showing that high-fat diets and diets enriched in both fat and refined sugars had, respectively, no effect [17,20] and inhibitory effects [21,22] on locomotion. In addition, while in prior studies the diets often resulted in increased BW or adipose mass in the offspring, piglets fed the HFS diet were not overweight in our study, indicating that the increase in BW induced by elevated levels of dietary fat may be the critical parameter required to observe decreased locomotion [22].

In addition to the independent effects of the prenatal or postnatal HFS diet, we found that several effects of the postnatal diet on behaviours depended on the prenatal diet. Piglets subjected to a switch of diet at birth spent less time inactive, and more time exploring and/or ingesting feeding materials than piglets maintained on the same treatment. Numerical data also suggest that piglets subjected to a switch of diet at birth spent more time exploring the environment, mounting and exploring pen mates than piglets maintained on the same diet. Mammals have the ability to perceive *in utero* the flavour of the maternal feed, which reaches the foetus through the amniotic fluid [49]. This ability enables the individual to identify, after birth, the organoleptic properties of their early postnatal diet that were already present (or not) in the maternal diet during gestation, which influences the development of food preferences [50,51]. We speculate that the disruption of this “*in utero-*early life sensory continuum” in piglets subjected to a dietary change at birth might have influenced their exploratory and social behaviour. Indeed, the switch of feed may have stimulated exploratory behaviours (to seek for the familiar prenatal diet), and social interactions (partly as re-directed exploratory behaviours). On the other hand, piglets that remained on the same diet may have been less motivated to seek for an alternative feed, and spent more time inactive. Although further studies would be needed to confirm this postulate, our findings emphasise the importance of the interactions between foetal and early life nutritional factors for the development of (eating) behaviours.

In our study, piglets exposed to the HFS diet *before birth* were heavier at weaning than control piglets, but this effect was not sustained after weaning. This confirms prior findings in rodents that a maternal high-fat diet increases offspring BW during the pre-weaning period, but not necessarily later [17]. Supplementing sow diets with fat during gestation has been found to affect both milk yield and composition [52,53], two limiting factors of piglet growth [54,55]. We therefore assume that the increase in piglet BW at weaning was induced by an increased milk production (and intake) and/or by changes in milk nutrient composition, *e*.*g*. elevated levels of fat [56]. Further research should include milk analyses to disentangle the respective role of milk composition and quantity on piglet BW at weaning.

The intake of the HFS diet *after birth* resulted in a 12% reduction in ADFI from weaning to the end of the treatment. The feed preference tests revealed consistent long- and short-term preferences for the HFS diet in piglets exposed to this diet *after birth*. Since piglets fed the control diet *after birth* did not show significant preferences for the control diet, we assume that the preferences for the HFS diet were not only due to habituation processes, but also to a high perceived palatability. Therefore, rather than from low palatability, the reduced ADFI in these piglets likely resulted from high satiating effects of the HFS diet, as confirmed by behavioural data showing that piglets fed the HFS diet *after birth* explored feed materials after weaning less than control pigs, *i*.*e*. showed fewer appetitive ingestive behaviours, indicating a lower motivation for feed [57], which may result from higher satiety. Accordingly, Besson *et al*. [58] found that minipigs were more motivated to obtain a HFS diet than a control diet at the very beginning of a progressive ratio task (*i*.*e*. higher palatability), but obtained fewer rewards in total at the end of the task (*i*.*e*. quicker satiating effect). In our study, piglets fed the HFS diet *after birth* had a lower intake of fibres and proteins, two nutrients known to enhance satiety [59,60], than control piglets, suggesting that other factors contributed to the dietary effects on ADFI. Energy intake appears to be a major determinant of feed intake in pigs [61], indicating that the lower ADFI found in piglets fed the HFS diet may simply result from a higher total energy intake (6.42 *vs*. 5.78 MJ NE/day).

Despite the higher caloric intake, piglets exposed to the HFS diet *after birth* weighed less at the end of the treatment than control piglets, which was caused by a 30% reduction in ADG from weaning onwards. Interestingly, G:F from weaning to the end of the dietary intervention was also lower in piglets fed the HFS diet *after birth* compared to control pigs. Crude protein [62] and amino acid (AA) levels, *e*.*g*. lysine [63], are known to be critical factors for (muscle) growth and feed efficiency in pigs. According to recommendations for weaned piglets by CVB [31], the HFS diet was deficient in essential AA, particularly lysine. In addition, piglets fed the HFS diet *after birth* ate significantly less than the control pigs from weaning (4 weeks of age) to 8 weeks of age, which resulted in a lower AA intake relative to net energy (*e*.*g*. lysine: 4.3 *vs*. 6.2 g/day). The decrease in growth and feed efficiency found in piglets fed the HFS diet *after birth* was thus likely caused by a lower intake and deposition of AA during the early post-weaning period, which the pigs apparently did not compensate for by increasing their feed intake. Another hypothesis to explain the reduced growth of piglets fed the HFS diet would be that piglets had difficulties to digest the high levels of animal fat contained in the HFS diet. However, although fat digestion has been reported to be limited in weaned piglets [64–66], the calculated NE intake was 11% higher in the piglets fed the HFS diet than in control piglets, indicating that a reduction in the intake of essential AA, such as lysine, rather than energy limited the growth rate of these piglets, and thus decreased feed efficiency.

We cannot exclude the possibility that some of the behavioural changes observed in piglets fed the postnatal HFS diet were caused by the reduction in BW gain, as a relationship between BW gain and agonistic behaviour around weaning and regrouping in piglets has been reported [67]. In our study, however, some behaviours were also affected by the interaction between prenatal and postnatal diet, suggesting that reduction in BW (gain) is likely not the only factor underlying the effects of the HFS diet on behavioural activity, as the prenatal diet did not affect BW (gain). Further research using pair-feeding groups of piglets of similar BW (gain) and fed HFS or control diets would enable to disentangle the respective role of diet composition and growth on behavioural activity.

A remarkable finding is the long-lasting effects of the postnatal HFS diet on piglet performance. During the 8 weeks following the dietary intervention, compared to control piglets, piglets fed the HFS diet *after birth* had a 20% decrease in ADFI, paired with a slight reduction in ADG, resulting in a higher G:F. The improved feed efficiency, associated with the attenuation of the effects on growth (7% *vs*. 30% reduction during the early post-weaning period), suggests that, when fed a commercial diet that meets their needs for nutrients, the piglets fed the HFS diet *after birth* were able to partially compensate for the growth reduction caused by AA restriction in early life, as previously reported [68,69]. The 8-week period, however, may not have been long enough to fully compensate. As BW mainly depends on muscle weight, one hypothesis to explain the long-term retardation of growth would be that the postnatal HFS diet affected myogenesis. Muscle weight depends on both myofibre number and myofibre size. While the number of myofibres is determined mainly before birth, postnatal myofibre development consists mainly of increase in fibre size, *i*.*e*. myofibre hypertrophy [70]. It is possible that low AA intake in piglets fed the HFS diet during the 4 first post-weaning weeks delayed hypertrophic muscle growth, resulting in long-lasting changes in body composition, with reduced muscle tissue and increased adipose tissue, contributing to the long-lasting reduction of BW. Additionally, in contrast to the control group, feed efficiency of piglets fed the postnatal HFS diet remained unchanged from the early post-weaning period to the 8-week period following the dietary intervention. This may indicate that piglets fed the HFS diet directly *after birth* compensate for myofibre size and hypertrophy when exposed to a commercial diet with adequate levels of AA, leading to an improved G:F ratio in these pigs.

Alternatively, it is possible that the intake of high levels of fat in the HFS diet during early life altered early life gut development and long-term gut functioning in the HFS piglets, thus complicating the transition to a standard diet later in life. Accordingly, de Souza *et al*. [65] reported that the intake of high dietary levels of fat after weaning increased and decreased lipase and amylase activity, respectively. They posited that these changes in the activity of enzymes involved in lipids and starch break down may impair the piglets’ ability to cope with a standard (high-starch low-fat) diet later in life, *i*.*e*. during the recovery period needed for enzyme levels to rebound. Further research should consider investigating the impact of the HFS diet on digestive enzyme activity in weaned piglets, and its potential role for long-term performance and behavioural development.

While partial compensation for growth and feed efficiency occurred, piglets fed the HFS diet *after birth* showed a large decrease in ADFI during the 8 weeks following the dietary intervention. One postulate to explain the drastic and long-lasting reduction of feed intake would be that 8-week early life exposure to the HFS diet resulted in long-lasting alterations of the satiety/appetite regulation system of the piglets. These effects might be mediated by long-lasting changes of circulating blood levels of appetite-regulating metabolites, such as leptin, ghrelin, insulin or glucose. Accordingly, we reported a significant decrease in fasting blood insulin levels during the 8-week period following the dietary intervention in piglets fed the postnatal HFS diet compared to control piglets [71]. Further analyses, however, should be done to support the postulate of altered appetite regulation.

## Conclusions

In conclusion, the present study provides evidence that foetal or early life exposure to a diet enriched in saturated fat, sucrose and cholesterol has subtle effects on behaviour of (non-obese) piglets, with reduced aggression and increased locomotion, without having apparent adverse effects on anxiety-related behaviours after weaning. The exposure to the HFS diet *after birth* also induced long-lasting changes in voluntary feed intake, growth and feed efficiency in piglets, which may have resulted from early post-weaning AA restriction. Our study also emphasises the importance of the interactions between *in utero* and early postnatal nutrition for the behavioural development of the offspring, as some behavioural effects of postnatal exposure to the HFS diet depended on the prenatal dietary history. Further studies are needed to determine which factors, such as maternal and offspring metabolic status, diet composition or digestive enzymatic activity, underlie the equivocal results found between our study and prior research in rodents. Considering the current socio-economic context characterised by the widespread availability of diet high in fat and refined sugars, the characterisation of dietary or metabolic factors underlying the effects of a perinatal high-fat high-sugar high-cholesterol diet on behaviour in pigs could have interesting applications in terms of dietary recommendations for perinatal nutrition in humans.

## Supporting Information

S1 Table Behaviours of piglets in the home pen after weaning. (PDF)

S2 Table Behaviours of piglets during the combined open field and novel object test. (PDF)

S3 Table Behaviours of piglets during the combined open field and novel object test. (PDF)
