# Supplementary material for: Perinatal Exposure to a Diet High in Saturated Fat, Refined Sugar and Cholesterol Affects Behaviour, Growth, and Feed Intake in Weaned Piglets
Source: PLoS One. 2016 May 18;11(5):e0154698. doi: 10.1371/journal.pone.0154698 (PMC4871475; doi:10.1371/journal.pone.0154698)
Supplement: S1 Table — (PDF) [file pone.0154698.s001.pdf]

S1 Table. Behaviours of piglets in the home pen after weaning.

Behaviour	Description
Inactive behaviours	
Lying	Lying on side or belly without performing any other described behaviour
Sitting/kneeling	Sitting or kneeling without performing any other described behaviour
Standing	Standing without performing any other described behaviour
Locomotion behaviours	
Walking	Walking without performing any other described behaviour
Feed-related behaviours	
Exploring feeding materials	Sniffing or touching feeder without sampling food, touching drinking nipple
Ingesting feeding materials	Eating or chewing food from feeder or spilled food on floor, drinking from water nipple or eating or chewing wood shavings on the floor
Exploratory behaviours	
Exploring environment	Sniffing, touching, scraping the leg, chewing or rooting (substrate on) floor, toys or any part of the pen, chewing
Social behaviours	
Aggressing pen mates	Head knocking, ramming or pushing a pen mate, with or without biting, piglets involved in a mutual fight
Manipulating pen mates	Rubbing belly of a pen mate with up and down movements of the snout (belly nosing), nibbling, sucking or chewing the tail, ear or any part of the body of a pen mate
Mounting pen mates	Standing on hind legs while having front legs on body of a pen mate
Exploring pen mates	Touching or sniffing any part of the body of a pen mate
Playing behaviours	
Social play	Pivoting, rolling, sliding, running around the pen with pen mate(s), gently ramming or nudging pen mates
Non-substrate play	Pivoting, rolling, sliding, running around the pen alone
Substrate play	Shaking object (straw, toys)
Others	
Maintenance	Rubbing body against objects or pen mates, scratching body with hind legs
Eliminating	Defecating or urinating
