# Supplementary material for: Perinatal Exposure to a Diet High in Saturated Fat, Refined Sugar and Cholesterol Affects Behaviour, Growth, and Feed Intake in Weaned Piglets
Source: PLoS One. 2016 May 18;11(5):e0154698. doi: 10.1371/journal.pone.0154698 (PMC4871475; doi:10.1371/journal.pone.0154698)
Supplement: S2 Table — (PDF) [file pone.0154698.s002.pdf]

S2 Table. Behaviours of piglets during the combined open field and novel object test.

Behaviour	Definition
Locomotion states	
Walking	Walks or runs in the arena. All 4 legs move or the pig turns around at the same spot without moving all 4 legs.
Standing alert	Stands motionless with head fixed (up or down) and ears upright. The pig is not performing any other described behaviour.
Standing	Stands with four paws on the floor.
Sitting/Kneeling	Sits or kneels on the floor.
Lying	Lies on side or belly.
Behavioural states	
Nosing floor	Explores the floor by sniffing, nosing, or licking. Rooting disc is either in contact or very close to surface.
Rooting floor	Explores the floor by rooting with the rooting disc, *i.e.* the rooting disc exerts some force.
Nosing walls	Explores walls of arena by sniffing, nosing, or licking it. Rooting disc is either in contact or very close to surface.
Rooting walls	Explores walls of arena by rooting it with the rooting disc, *i.e.* the rooting disc exerts some force.
Approaching bucket, slow	Approaches the bucket slowly (step by step) within 1m distance, but does not explore it (yet).
Approaching bucket, fast	Approaches the bucket quickly (easy walking or running) within 1m distance, but does not explore it (yet).
Withdrawing from bucket	Draws back from the bucket. At first the head is directed to the bucket, during the draw back the head can be either directed to the bucket or turned away from it.
Exploring bucket	Noses or sniffs the metal bucket without touching it. Rooting disc is not in contact with the bucket.
Touching bucket	Noses, sniffs, roots or licks the metal bucket. Rooting disc is in contact with the bucket.
Chewing bucket	Tries to chew on the metal bucket by touching it with open mouth and making biting movements.
Vocalizations events	
Short grunt	A low tone of less than half a second (one note)
Long grunt	A low tone of more than half a second (one note)
Squeal	A high tone (different notes)
Grunt-squeal	A low tone that transforms into a high tone
Scream	A high, long and loud tone, often as long as an expiration
Bark	A low tone that sounds like “wuff”
Events: Other behavioural events	
Eliminating	Defecates and urinates
Maintenance	Rubs body against walls, scratches body with hind leg or stretches (part of) body.
Jumping	Jumps in air or against wall of the test arena trying to escape the arena.
