# Supplementary material for: Perinatal Exposure to a Diet High in Saturated Fat, Refined Sugar and Cholesterol Affects Behaviour, Growth, and Feed Intake in Weaned Piglets
Source: PLoS One. 2016 May 18;11(5):e0154698. doi: 10.1371/journal.pone.0154698 (PMC4871475; doi:10.1371/journal.pone.0154698)
Supplement: S3 Table — (PDF) [file pone.0154698.s003.pdf]

S3 Table. Behaviours of piglets during the combined open field and novel object test.

Prenatal diet	Control		HFS	
Postnatal diet	Control	HFS	Control	HFS
Open field test				
Walking (%)	63.0 ± 3.46	63.5 ± 2.74	69.7 ± 4.04	63.2 ± 2.15
Standing alert (%)	15.5 ± 2.65	19.0 ± 2.76	9.87 ± 3.21	15.9 ± 2.87
Standing (%)	21.4 ± 2.25	17.4 ± 2.78	19.2 ± 2.40	18.5 ± 2.56
Exploring arena (%)	67.2 ± 4.21	70.3 ± 3.45	78.1 ± 4.69	71.3 ± 3.39
Low-pitched vocalisations (freq)	65.1 ± 10.8	54.2 ± 12.9	48.4 ± 10.7	61.7 ± 10.5
High-pitched vocalisations (freq)	10.4 ± 5.27	7.50 ± 4.86	6.94 ± 3.00	3.56 ± 1.43
Eliminating (freq)	2.80 ± 0.54	2.06 ± 0.52	2.06 ± 0.52	1.63 ± 0.51
Distance covered (m)	8122 ± 837	9230 ± 732	8936 ± 921	9748 ± 715
Latency to centre zone (s)	37.9 ± 18.6	20.2 ± 10.1	5.16 ± 2.23	8.14 ± 4.05
Time in the centre zone (%)	14.9 ± 2.15	22.1 ± 2.84	19.5 ± 2.36	22.8 ± 2.73
Time in wall zone (%)	74.6 ± 3.13	67.7 ± 2.51	73.4 ± 1.96	69.5 ± 3.01
Time in observer zone (%)	47.9 ± 4.26	40.5 ± 3.51	44.9 ± 2.61	42.4 ± 3.33
Time in entrance zone (%)	12.0 ± 1.75	13.7 ± 2.22	12.8 ± 1.37	14.9 ± 1.30
Novel object test				
Walking (%)	40.9 ± 4.17	41.1 ± 5.24	46.9 ± 4.14	39.6 ± 3.72
Standing alert (%)	28.7 ± 4.19	37.3 ± 5.15	31.7 ± 3.10	35.0 ± 4.34
Standing (%)	23.2 ± 3.19	21.5 ± 2.73	19.4 ± 2.73	24.7 ± 2.83
Exploring arena (%)	23.1 ± 3.49	30.3 ± 4.23	34.4 ± 4.05	28.5 ± 4.83
Low-pitched vocalisations (freq)	50.7 ± 9.55	46.3 ± 9.21	49.9 ± 7.51	57.4 ± 8.28
High-pitched vocalisations (freq)	3.07 ± 1.98	2.19 ± 1.80	3.56 ± 1.65	3.69 ± 2.04
Eliminating (freq)	1.87 ± 0.42	1.56 ± 0.30	1.75 ± 0.36	1.06 ± 0.31
Distance covered (m)	5458 ± 673	4981 ± 583	5801 ± 645	4653 ± 554
Latency to centre zone (s)	11.8 ± 4.02	24.7 ± 16.2	10.4 ± 2.65	11.5 ± 8.30
Time in the centre zone (%)	34.4 ± 6.75	26.9 ± 5.67	34.9 ± 5.88	35.0 ± 5.85
Latency to novel object zone (s)	48.1 ± 18.2	53.0 ± 15.4	50.1 ± 15.5	71.9 ± 21.4
Time in novel object zone (%)	24.4 ± 6.22	16.9 ± 4.84	20.5 ± 5.02	19.7 ± 5.03
Time in wall zone (%)	55.1 ± 5.75	62.5 ± 6.77	55.2 ± 4.56	47.7 ± 6.32
Time in observer zone (%)	35.4 ± 6.19	25.5 ± 5.61	32.8 ± 4.85	22.8 ± 4.83
Time in entrance zone (%)	14.8 ± 5.47	6.01 ± 1.26	6.80 ± 1.84	8.13 ± 1.93
Approaching bucket (%)	5.71 ± 1.16	4.68 ± 0.96	6.23 ± 1.02	4.99 ± 0.65
Withdrawing from bucket (%)	2.69 ± 0.89	1.77 ± 0.46	3.33 ± 0.57	1.68 ± 0.33
Exploring bucket (%)	13.4 ± 3.85	9.89 ± 3.05	11.2 ± 2.58	16.9 ± 4.18

Data are presented as means ± SEMs.
